# Management of Ptosis in Kearns–Sayre Syndrome: A Case Report and Literature Review

**DOI:** 10.1055/a-2207-7587

**Published:** 2024-04-08

**Authors:** Moulay O. Moustaine, Zakaria Azemour, Frarchi Mohammed, Othman Benlanda, Hicham Nassik, Mehdi Karkouri

**Affiliations:** 1 Department of Ophthalmology, CHU Souss Massa, Faculty of Medicine and Pharmacy, Ibn Zohr University, Agadir, Morocco; 2 Department of Anesthesia Resuscitation, CHU Souss Massa, Faculty of Medicine and Pharmacy, Ibn Zohr University, Agadir, Morocco; 3 General Laboratory of Pathological Anatomy, CHU Ibn Rochd, Faculty of Medicine and Pharmacy, Hassan II University, Casablanca, Morocco

**Keywords:** Kearns–Sayre syndrome, ptosis, chronic progressive external ophthalmoplegia, Frontal suspension

## Abstract

Kearns–Sayre syndrome (KSS) is a rare mitochondrial disease that affects young adults, due to a deletion of mitochondrial DNA and characterized by the triad: age of onset lower than 20 years, chronic progressive external ophthalmoplegia, and an atypical pigmentary retinopathy. It is also characterized by other endocrine, neurological, and especially cardiac impairment with a very high risk of cardiac complications during surgical procedures under all types of anesthesia.

We report a case of KSS revealed by severe bilateral ptosis and confirmed by a muscle biopsy with “ragged red fibers.” The ptosis was surgically managed by cautious Frontal suspension under local anesthesia “Frontal nerve block.” Through this case, we discuss challenges in the management of KSS patients.

## Introduction

Kearns–Sayre syndrome (KSS) is a rare mitochondrial disease that affects young adults and is caused due to a deletion of mitochondrial DNA. 1 It is defined by the following triad: onset before the age of 20, chronic progressive external ophthalmoplegia, and pigmentary retinopathy. 2 3 Given the frequency of cardiac conduction defects, there is a very high risk of cardiac complications, especially during surgical procedures under general anesthesia.

We report a case of KSS revealed by severe bilateral ptosis that was surgically managed in our ophthalmology department.

## Case

A 16-year-old male, with no past medical history, no inbreeding or similar cases in the family, consulted our ophthalmology department for a gradual dropping of both upper eyelids during the past 5 years with hemeralopia, without fluctuation or triggering factors.

**Fig. 1.**
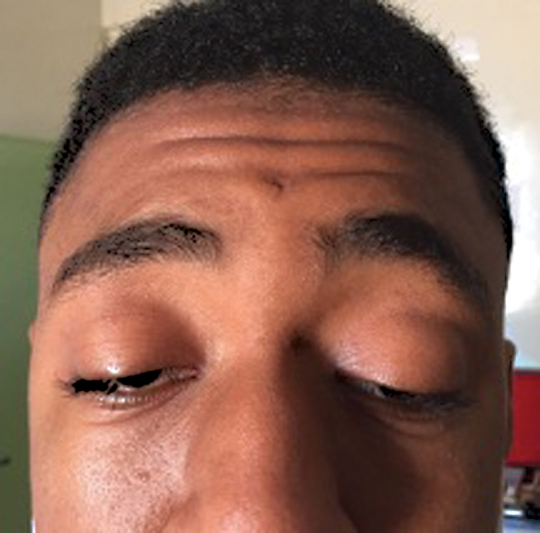
Bilateral severe ptosis more marked in the left eye with forehead wrinkling.

Ophthalmological examination found a severe bilateral ptosis—more marked in the left eye with a palpebral slit at 5 and 7 mm in the right eye; the action of the upper eyelid levator was 5 mm in both eyes with hyper action of the frontal muscle with forehead wrinkling ( Fig. 1 ). Eyelid crease was absent from the left eye and located at 12 mm in the right eye.

**Fig. 2.**
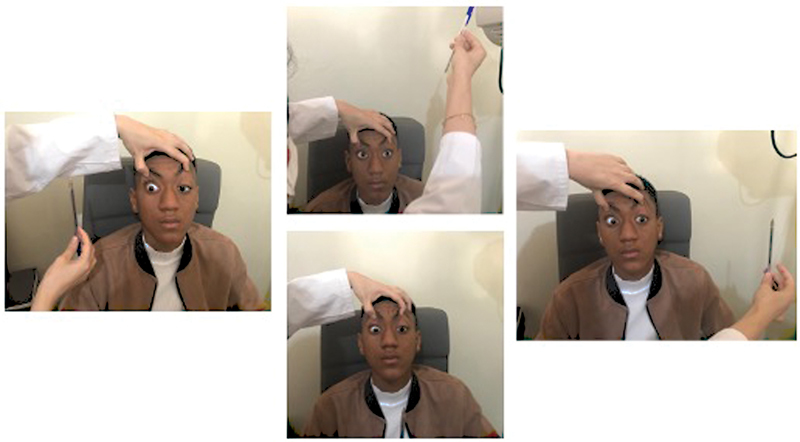
Bilateral external ophthalmoplegia.

The evaluation of ocular motility revealed a bilateral ophthalmoplegia with negative Charles Bell sign ( Fig. 2 ). Direct and consensual photomotor reflexes were normal.

**Fig. 3.**
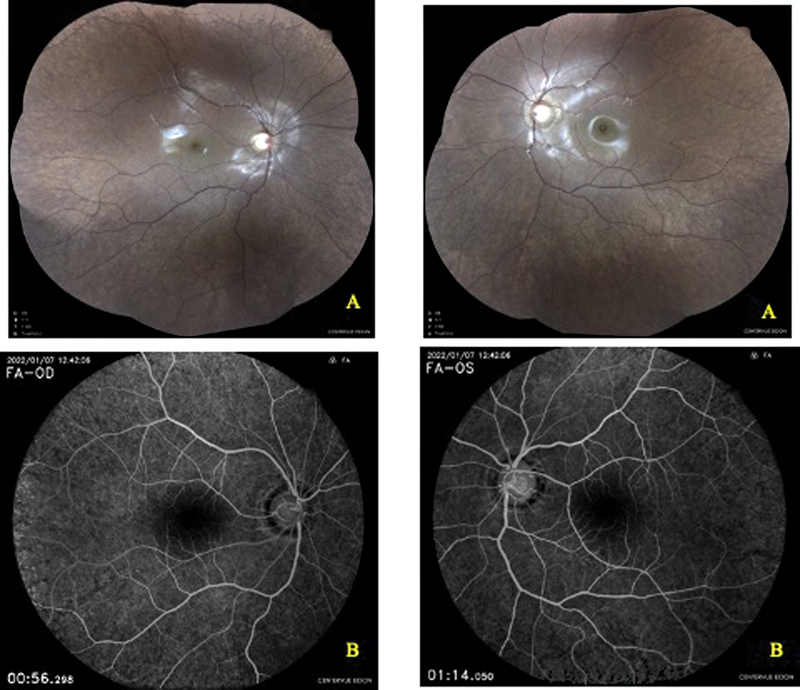
Fundus ( **A** ) and fluorescein angiography photography ( **B** ) showed an atypical pigmentary retinopathy with a “salt and pepper” appearance.

Visual acuity was 10/10-P2 in the right eye, and 8/10-P2 in the left eye. At slit-lamp examination, anterior segment was normal with intraocular pressure at 12 mm Hg in both eyes. Fundus examination showed an atypical pigmentary retinopathy ( Fig. 3 ). Extraocular muscle assessment was normal and the phenylephrine test was negative.

Parents also reported an increasingly evident cognitive and concentration impairment. However, the cardiologic, neurological, and general examinations were normal.

Retinal fluorescein angiography revealed a “salt and pepper” appearance with areas of hyper- and hypofluorescence. A brain MRI was done to search a possible intracranial process revealed a bilateral exophthalmos grade I, without hypertrophy of the ocular muscles or periorbital adipose tissue.

The diagnosis of KSS was highly suspected given the young age of the patient (16 years) and the association of progressive ptosis, external ophthalmoplegia, and the appearance of atypical retinitis pigmentosa.

**Fig. 4.**
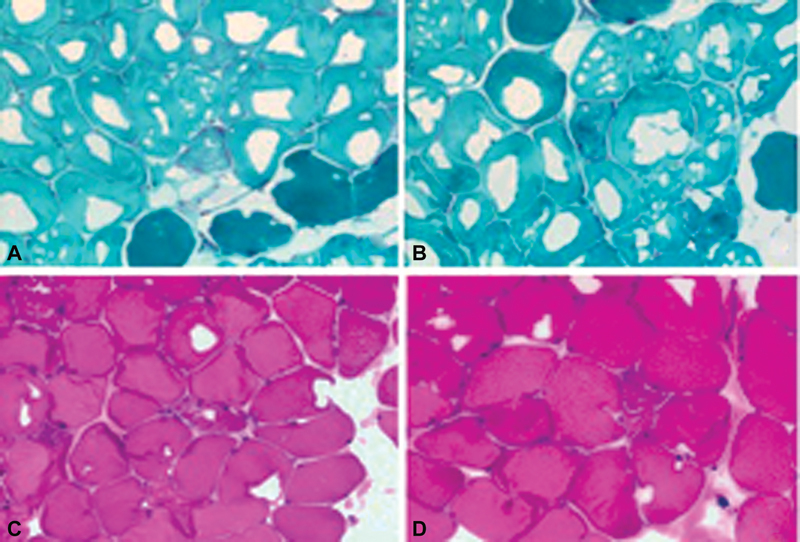
Biopsy of deltoid muscle reveals. ( **A** ) “Ragged red fibers” on Gomori staining (grossissement × 40). ( **B** ) Muscle fibers with a defective cytochrome C oxidative activity on coloration hematein–eosin (grossissement × 40).

A biopsy of skeletal muscle (deltoid muscle) confirmed the diagnosis of KSS, by showing ragged red fibers that stain red using trichrome stain associated with a defective cytochrome C oxidative activity on coloration hematein–eosin ( Fig. 4 ).

**Fig. 5.**
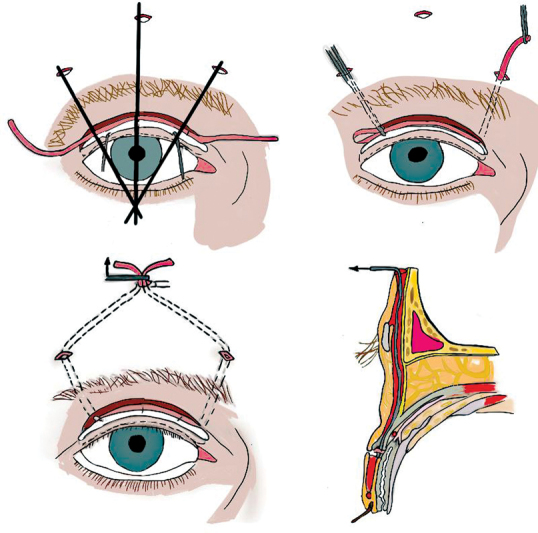
Palpebral suspension using the Fox procedure.

In therapeutic terms, our patient received a medical treatment based on the coenzyme 10 (cofactor of the respiratory chain). For the ptosis, our management was a partial and cautious suspension to the frontalis muscle of the two levators of the upper eyelids using the Fox procedure ( Fig. 5 ) to clear the pupillary center (visual axis).

We opted for a ptosis surgery under local anesthesia “Frontal nerve block” using a mix of Bupivacaine (2.5 mg/mL) and Xylocaine (10 mg/ml), to avoid complications of general anesthesia.

In Fox procedure, we begin by making three palpebral incisions, each 2 mm long, 2 mm above the ciliary line, followed by two deeper incisions at the upper edge of the eyebrow, and a final incision in the forehead, 10 mm above the eyebrow. Together, these incisions give the suspension loop a pentagonal shape. A 3/0 polypropylene wire is threaded through the loop, with the help of a metal guide that tunnels the loop behind the orbicularis muscle. The desired amount of suspension is obtained by crossing and pulling the two heads of wire.

A second 4/0 polypropylene wire is used to fix and lock the two heads of the first wire. The frontal subcutaneous plane is then sutured with 6/0 absorbable thread.

**Fig. 6.**
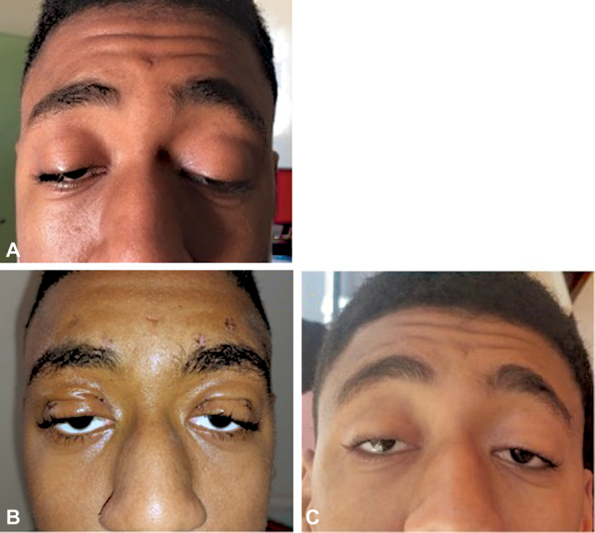
( **A** ) Preoperative appearance. ( **B** ) Appearance, 5 days after surgery. ( **C** ) Appearance, 3 months after surgery.

Postop eye occlusion was done using a suspension of the lower eyelid with steri-strips for 2 weeks associated with artificial tears application to prevent the corneal damage by exposure. The final result was satisfactory ( Fig. 6 ).

## Discussion

KSS is a rare neuromuscular disease due to a mitochondrial cytopathy, described for the first time by Thomas Kearns and George Pomeroy Sayre in 1958. 4

The majority of KSS cases are sporadic, with the most common deletion labeled as the “common 4977-bp deletion;” 3 5 however, in about 15% of cases, the transmission is autosomal dominant or recessive, with no risk factors or predilection for sex or race. 6

Clinically, KSS is a heterogeneous neurodegenerative syndrome involving the musculoskeletal, central nervous, cardiovascular, and endocrine systems.

The ophthalmological abnormalities are in the foreground. Chronic progressive external ophthalmoplegia that affects both ocular muscles and eyelid levator muscles resulting in a severe ptosis which is most often the revealing sign (46% of cases). 7 Pigmentary retinopathy is atypical, 8 9 which was the case for our patient who had a preserved visual acuity and a late hemeralopia without papillary atrophy or narrowing of the arteries caliber.

However, the exophthalmos without hypertrophy of the ocular muscles or periorbital adipose tissue noted in our case is very rare. It is assumed that is due to muscle relaxation which leads to a protrusion of the eyeball.

At the extra-ophthalmological level, cardiomyopathy is present in 57% of cases responsible for cardiac conduction disorders causing arrhythmia or a cardiac embolus which are the most serious cardiovascular manifestations leading to sudden death in 11% of patients. 3 7

Central nervous and endocrine disorders could be frequently observed: ataxia, hyperproteinorachia, peripheral neuropathy, deafness, cognitive deficit, muscle weakness, diabetes insipidus, hypoparathyroidism, growth hormone deficiency, hypogonadism, and renal tubular acidosis that occasionally progress to end-stage renal failure. 6 7

MRI can be useful in revealing a possible leukoencephalopathy in patients with central nervous system involvement by showing hyperintensities on fluid-attenuated inversion recovery sequences in the brainstem, globus pallidus, thalamus, and white matter of the cerebrum and cerebellum. 3

The diagnosis of a KSS is confirmed by muscle biopsy that shows an appearance of “ragged red fibers” that stain red or purple using a modified Gomori trichrome stain with cytochrome C oxidase-negative. 10 There is also an accumulation of abnormal mitochondria in the subsarcolemma and an increase in muscle enzymes like phosphokinase and lactates. 11

The ptosis poses enormous problems in KSS patients given the ophthalmoplegia and the absence of Charles Bell phenomenon.

On the one hand, the indication for surgery is reversed, thus for some authors, surgical management of ptosis is deconsolidated, given the risk of exposure keratitis. However, a partial suspension of upper eyelid to frontalis muscle is indicated if necessary to expose visual axis. The amount of ptosis correction should be limited with the aimed at clearing the pupillary center (visual axis) and not a total correction of the ptosis, in order to avoid the risk of corneal damage by exposure, also eye occlusion and eye lubricants are recommended to avoid corneal complications.

On the other hand, the peroperative ptosis management of KSS is a real challenge for the anesthetist, given the high risk of complications linked to cardiac conduction disturbances, malignant hyperthermia, respiratory complications, and hypoglycemia.

For this reason, locoregional anesthesia is preferable, given the reduced risk of complications. It should be preferred to optimize surgery management. A frontal nerve block, was used to manage the ptosis of our patient. This nerve bloc is generally useful for upper eyelid surgery such as repair of ptosis; it allows a block of the frontal nerve's dividing branches (supratrochlear, lateral, and medial supraorbital).

General anesthesia should be avoided as much as possible in order to limit the use of halogenated agents that can cause malignant hyperthermia. 12 13 In the event that general anesthesia is unavoidable, the use of total intravenous anesthesia with propofol, fentanyl, and alfentanil is preferable with minimal doses of neuromuscular blocker agents. 12 13 14

Whatever the type of anesthesia, it is necessary to monitor heart rate and respiratory parameters, maintain normothermia and normoglycemia. 14 15

Because of the diversity of the symptoms presented by KSS patients, the management must be multidisciplinary. Currently, there is no effective treatment, the most used treatment is the mitochondrial antioxidant CoQ 10 (ubiquinone), with no studies proving its actual benefit. 3 16 17 Our patient was given subcoenzyme Q10 for a year, but without any improvement.

### Conclusion

There are many challenges in the management of KSS. Firstly, there is no proven medical treatment up to now. Secondly, the surgical management of ptosis is controversial, thus surgery indications are limited to the cases where the ptosis affects the visual function in the aim to expose the visual axis. Thirdly, general anesthesia should be avoided as far as possible in favor of cautious locoregional anesthesia with a good heart examination to detect and manage conducting cardiac disorders.

In the future, with gene therapy research, we hope to have a potential treatment which may attempt to inhibit mutant mtDNA replication or encourage replication of wild-type mtDNA.
